# Supplementary material for: Flux analysis of cholesterol biosynthesis in vivo reveals multiple tissue and cell-type specific pathways
Source: eLife. 2015 Jun 26;4:e07999. doi: 10.7554/eLife.07999 (PMC4501332; doi:10.7554/eLife.07999)
Supplement: Supplementary file 1. — Rate constants (k) and concentrations of cholesterol biosynthetic intermediates in mouse tissues. DOI: http://dx.doi.org/10.7554/eLife.07999.012 [file elife07999s001.docx]

|  | **Testis** | | **Spleen** | | **Adrenal** | | **BAT** | | **WAT** | | **Kidney** | |
| --- | --- | --- | --- | --- | --- | --- | --- | --- | --- | --- | --- | --- |
|  | K^#^ | Conc. [ng/mg] | k | Conc. [ng/mg] | k | Conc. [ng/mg] | k | Conc. [ng/mg] | k | Conc. [ng/mg] | k | Conc. [ng/mg] |
| Lanosterol | 0.05488 | 134 | 0.11000 | 25 | 0.49670 | 49 | 0.11280 | 17 | 0.14370 | 14 | 0.37200 | 27 |
| Dihydrolanosterol | 0.00031 | 5 | 0.00698 | 5 | 0.02017 | 34 | 0.00501 | 7 | 0.01788 | 3 | 0.09780 | 3 |
| Dihydro-ff-MAS | 0.00007 | 32 | 0.00780 | 3 | 0.00876 | 18 | 0.00010 | 0 | 0.00193 | 2 | 0.03606 | 2 |
| Dihydro-t-MAS | 0.00330 | 21 | 0.00000 | 9 | 0.04380 | 10 | 0.01041 | 2 | 0.02066 | 5 | 0.06780 | 3 |
| 7-Dehydrocholesterol | 0.00029 | 41 | 0.00860 | 24 | 0.15780 | 17 | 0.08340 | 5 | 0.11340 | 4 | 0.09749 | 31 |
|  |  |  |  |  |  |  |  |  |  |  |  |  |
| Lanosterol | 0.05488 | 134 | 0.11000 | 25 | 0.49670 | 49 | 0.11280 | 17 | 0.14370 | 14 | 0.37200 | 27 |
| ff-MAS | 0.17650 | 40 | 0.62400 | 8 | 1.55600 | 13 | 0.27561 | 8 | 0.08577 | 14 | 1.62400 | 3 |
| t-MAS | 0.00872 | 948 | 0.21053 | 17 | 0.43650 | 63 | 0.16410 | 11 | 0.10680 | 24 | 1.30530 | 8 |
| Zymosterol | 0.02313 | 92 | 0.14370 | 19 | 2.64300 | 11 | 0.64440 | 2 | 1.10670 | 4 | 1.24370 | 5 |
| Dehydrodesmosterol | 0.03398 | 61 | 0.14880 | 22 | 1.23500 | 17 | 0.26580 | 7 | 0.29580 | 16 | 2.62880 | 2 |
| Desmosterol | 0.00398 | 606 | 0.14390 | 18 | 0.20450 | 113 | 0.04410 | 48 | 0.02868 | 64 | 0.44390 | 17 |
|  | | | | | | | | | | | | |
|  | **Liver** | | **Heart** | | **Muscle** | | **Brain** | | **Skin** | | **Preputial** | |
|  | k | Conc. [ng/mg] | k | Conc. [ng/mg] | k | Conc. [ng/mg] | k | Conc. [ng/mg] | k | Conc. [ng/mg] | k | Conc. [ng/mg] |
| Lanosterol | 0.45200 | 29 | 0.02340 | 16 | 0.00947 | 32 | 0.03172 | 4 | 0.32350 | 23 | 0.04154 | 75 |
| Dihydrolanosterol | 0.12320 | 17 | 0.00243 | 6 | 0.00064 | 5 | 0.01242 | 2 | 0.05960 | 10 | 0.00060 | 168 |
| Dihydro-ff-MAS | 0.00214 | 23 | 0.00015 | 2 | 0.00001 | 3 | 0.03606 | 1 | 0.06430 | 4 | 0.00019 | 26 |
| Dihydro-t-MAS | 0.02263 | 45 | 0.00487 | 13 | 0.00840 | 7 | 0.01734 | 2 | 0.68879 | 8 | 0.01692 | 35 |
| 7-Dehydrocholesterol | 0.21880 | 25 | 0.01140 | 29 | 0.00678 | 43 | 0.02275 | 4 | 0.86400 | 12 | 0.00521 | 748 |
|  |  |  |  |  |  |  |  |  |  |  |  |  |
| Lanosterol | 0.25200 | 49 | 0.02340 | 16 | 0.00947 | 32 | 0.03172 | 4 | 0.32350 | 23 | 0.04154 | 75 |
| ff-MAS | 0.36133 | 51 | 0.17480 | 2 | 0.05424 | 8 | 0.06240 | 3 | 0.18589 | 33 | 0.09293 | 28 |
| t-MAS | 0.31200 | 47 | 0.01720 | 17 | 0.02470 | 15 | 0.03053 | 7 | 0.13560 | 50 | 0.20137 | 17 |
| Zymosterol | 0.21800 | 42 | 0.05740 | 5 | 0.01678 | 15 | 0.24370 | 1 | 0.05689 | 19 | 0.25816 | 7 |
| Dehydrodesmosterol | 0.26867 | 29 | 0.02470 | 7 | 0.00845 | 9 | 0.00629 | 7 | 0.09860 | 11 | 0.01036 | 19 |
| Desmosterol | 0.23267 | 29 | 0.01443 | 17 | 0.00327 | 51 | 0.00074 | 37 | 0.00660 | 326 | 0.00233 | 111 |

**Supplementary File 1:** Rate constants (k) and concentrations of cholesterol biosynthetic intermediates in mouse tissues.

#- Rate constants were determined by fitting fractional deuterium labeling at each time point to the first order kinetic model.
g=1-e^-kt^ using the Matlab curving fitting toolbox. Rate constants, as shown in the main figure, were determined by multiplying
k and concentration. See **Methods** for more details
