# Supplementary material for: Flux analysis of cholesterol biosynthesis in vivo reveals multiple tissue and cell-type specific pathways
Source: eLife. 2015 Jun 26;4:e07999. doi: 10.7554/eLife.07999 (PMC4501332; doi:10.7554/eLife.07999)
Supplement: Supplementary file 2. — LC-MS/MS characteristics of sterols. DOI: http://dx.doi.org/10.7554/eLife.07999.013 [file elife07999s002.docx]

**Supplementary File 2: LC-MS/MS Characteristics of Sterols**

| Sterol | Retention Time [min] | m/z [Da] | Isotopomer Range |
| --- | --- | --- | --- |
| Lanosterol | 7.0 | 409 | 409-412 |
| Dihydrolanosterol | 8.1 | 411 | 411-414 |
| ff-MAS | 6.1 | 393 | 393-396 |
| Dihydro-ff-MAS | 6.8 | 395 | 395-398 |
| t-MAS | 7.3 | 395 | 395-398 |
| Dihydro-t-MAS | 8.4 | 397 | 397-400 |
| Zymosterol | 5.6 | 367 | 367-368 |
| Zymostenol | 6.3 | 369 | N.D. |
| Dehydrolathosterol | 5.4 | 367 | N.D. |
| Lathosterol | 6.6 | 369 | N.D. |
| Dehydrodesmosterol | 5.0 | 365 | 365-368 |
| 7-Dehydrocholesterol | 6.4 | 367 | 367-368 |
| Desmosterol | 5.9 | 367 | 367-368 |
| Cholesterol | 6.8 | 369 | Saturated Signal |
| d6-Sitosterol | 7.4 | 404 | 404 |

Mass spectral and chromatographic parameters of the sterols measured in this study. Sterols in the cholesterol biosynthetic pathways were measured using LC-MS/MS and isotopic labeling. Sterols were resolved with a Shimadzu LC20 HPLC on a C18 column with a linear gradient transitioning from 7% H_2_O/93% methanol to 100% methanol over 7 min, then maintaining 100% methanol for 5 min. The retention times for each sterol analyzed are provided. The sterols and their isotopes were measured using an ABSciex Q-Trap 4000. The mass to charge ratio (m/z) of the monoisotopic peak of each sterol and the range analyzed during flux analysis were measured using MRM pair with identical MS1 and MS2 masses. Zymostenol, dehydrolathosterol, and lathosterol were not detectable for analysis by D_2_O labeling. Cholesterol is >1000 times more abundant than any of the sterol intermediates so the levels exceeding the linear range for flux analysis with other sterols. d_6_-sitosterol was used as an internal standard in all experiments for quantification.
