# Supplementary material for: Flux analysis of cholesterol biosynthesis in vivo reveals multiple tissue and cell-type specific pathways
Source: eLife. 2015 Jun 26;4:e07999. doi: 10.7554/eLife.07999 (PMC4501332; doi:10.7554/eLife.07999)
Supplement: Supplementary file 3. — Primer sequences for real-time PCR of genes in the cholesterol biosynthetic pathway. DOI: http://dx.doi.org/10.7554/eLife.07999.014 [file elife07999s003.docx]

**Supplementary File 3**. Primer sequences for real-time PCR of genes in the cholesterol biosynthetic pathway.

| Species | **Gene** | **Sequence (5' -> 3')** |
| --- | --- | --- |
| Human | HMGCR | CAAGGAGCATGCAAAGATAATCC GCCATTACGGTCCCACACA |
| Human | SQLE | GAGATGGAAGAAAGGTGACAGTCA CACCCGGCTGCAGGAAT |
| Human | LSS | CGCTCAACTATGTGTCTCTCAGAATT TGTTCCGGGCTCGTACCA |
| Human | CYP51A | GCAGGGATGCTTATTGGATTACTC AGAAGCCCATCCAAGCACTAG |
| Human | LBR | CAAAAGAACTGGCAGTGAGAACCT ACTCCTCCAAACTCCAAGTCCTT |
| Human | TM7sf2 | TGAAGGAGGCAGAGCTTCGA ACCCACGTAGAGCAACTGGAA |
| Human | SC4MOL | AGAAAAGCCGGCACCAAGA TCAAAGAGAGAATCAGCTCAAACTG |
| Human | NSDHL | CCCAGGTGCGGTTCTTTCT TACACCTTTCAGAGCTGGGTACAG |
| Human | HSD17B7 | ATGGAACAGAAGCTCTGGTATGG GTGGTGGCACTCAGATATTTGATC |
| Human | EBP | GCCCACAAAGACATGACTACCA GGCGGTCATTAGGTACAAAGTTG |
| Human | SC5D | GGGCACTGCTGAGGAATGAT CAACATCTGGCATTTTAAGCATAAG |
| Human | DHCR7 | ACGCTACACTCTGCCCACACT CAAGAGTAAATGCAGCCTAATGACA |
| Human | DHCR24 | GCGGGACATCCAGAAGCA CCGTGCACATGAAGGTCTTG |
| Human | 36B4 | GGCCTGAGCTCCCTGTCTCT GCGGTGCGTCAGGGATT |
|  |  |  |
| Mouse | DHCR24 | AGGCAGCTGGAGAAGTTTGTG CCTCGCGGTTCATATAGCAATC |
| Mouse | 36B4 | CACTGGTCTAGGACCCGAGAAG GGTGCCTCTGGAGATTTTCG |
